# Gross morphology, histology, and ultrastructure of the olfactory rosette of a critically endangered indicator species, the Delta Smelt, *Hypomesus transpacificus*

**DOI:** 10.1007/s00359-021-01500-7

**Published:** 2021-06-22

**Authors:** Pedro Alejandro Triana-Garcia, Gabrielle A. Nevitt, Joseph B. Pesavento, Swee J. Teh

**Affiliations:** 1 Integrative Pathobiology Graduate Group and Aquatic Health Program, Department of Anatomy, Physiology and Cell Biology, School of Veterinary Medicine, University of California, VM3B, 3203, 1089 Veterinary Medicine Dr, Davis, CA 95616 USA; 2 Grupo de Investigación en Sanidad de Organismos Acuáticos, Instituto de Acuicultura de Los Llanos, Universidad de Los Llanos, Villavicencio, Meta Colombia; 3 Department of Neurobiology, Physiology and Behavior, University of California, Davis, CA USA; 4 California Animal Health and Food Safety Laboratory, School of Veterinary Medicine, University of California, Davis, CA USA

**Keywords:** Sensory ecology, Immunohistochemistry, Olfaction, Fish, Neuroanatomy

## Abstract

The Delta Smelt (*Hypomesus transpacificus*) is a small, semi-anadromous fish native to the San Francisco Bay-Delta Estuary and has been declared as critically endangered. Their olfactory biology, in particular, is poorly understood and a basic description of their sensory anatomy is needed to advance our understanding of the sensory ecology of species to inform conservation efforts to manage and protect them. We provide a description of the gross morphology, histological, immunohistochemical, and ultrastructural features of the olfactory rosette in this fish and discuss some of the functional implications in relation to olfactory ability. We show that Delta Smelt have a multilamellar olfactory rosette with allometric growth. Calretinin immunohistochemistry revealed a diffuse distribution of olfactory receptor neurons within the epithelium. Ciliated, microvillous and crypt neurons were clearly identified using morphological and immunohistochemical features. The olfactory neurons were supported by robust ciliated and secretory sustentacular cells. Although the sense of smell has been overlooked in Delta Smelt, we conclude that the olfactory epithelium has many characteristics of macrosmatic fish. With this study, we provide a foundation for future research into the sensory ecology of this imperiled fish.

**Supplementary Information:**

The online version contains supplementary material available at 10.1007/s00359-021-01500-7.

## Introduction

Comparative anatomy of fish olfactory systems was established as a field of study more than a century ago when one of the most thorough reviews was published by Burne (1909). This remarkable paper was the first to highlight the astonishing diversity of fish olfactory systems. Since Burne, the field has grown rapidly to elucidate the complexity and diversity of fish olfaction, and how basic design features are evolutionarily well conserved among fish and other vertebrates (Kasumyan 2004; Hansen and Zielinski 2005). This comparative approach led researchers to conclude that structural morphology can predict ecological adaptations (Kasumyan 2004; Schluessel et al. 2008). For example, some fish species have been classified as macrosmatic or microsmatic, depending on the morphological characteristics of their olfactory systems (Atta 2013).

In general, the fish olfactory system is composed of a pair of peripheral multilamellar sensory organs called the olfactory rosettes. The axons of sensory neurons in the rosette make up the olfactory nerve, and form synapses in the olfactory bulbs. In many teleost species that have been studied (for review, see Satou 1990; Zielinski and Hara 2006), each olfactory bulb is composed of four distinctive concentric layers referred to, from periphery to center, as the olfactory nerve layer, the glomerular layer, the mitral cell layer and the granular cell layer. The olfactory nerve fascicles form the olfactory nerve layer and connect the peripheral sensory neurons to primary output neurons (i.e., the mitral cells and the ruffed cells) in the olfactory bulb. The synapses between the olfactory neurons and the primary output neurons in the bulb form spherical structures composed of neuropil called glomeruli which together form the glomerular layer. The mitral cell layer and granular cell layers are formed by mitral cells which are primary output neurons, and granule cells, which are interneurons that synapse mainly with mitral cells (Zielinski and Hara 2006). The olfactory bulbs connect to higher brain centers via the olfactory tracts (Hamdani and Døving 2007; Hara 2011).

The olfactory rosette is made up of multiple leaf-like structures that contain a variety of functionally specific cell populations. The olfactory epithelium in a macrosmatic fish is composed of millions of olfactory neurons (Easton 1971; Kreutzberg and Gross 1977), embedded between sustentacular cells and ciliate cells that support them. A wide diversity of olfactory neurons has been described in fishes (Hansen and Zielinski 2005), including ciliated olfactory sensory neurons (ciliated OSNs) and microvillous neurons. Recently other neuronal cell types have been described, including crypt neurons, the Kappe neurons (Hansen and Finger 2000; Ahuja et al. 2014) and the pear-shaped neurons (Wakisaka et al. 2017). Different experimental approaches have been developed to characterize the morphology and function of these sensory neurons, ranging from fundamental histological (Ferrando et al. 2016), ultrastructural (Bannister 1965), and immunohistochemical methods (Ferrando et al. 2009) to whole transcriptome sequencing (Fatsini et al. 2016).

Functionally, the olfactory neurons have specific olfactory receptors that are tuned to detect specific types of odorant ligands (Bazaes et al. 2013). The ciliated olfactory neurons are characterized by having a long dendrite with an olfactory knob crowned by several sensory cilia (Bannister 1965). These cells express olfactory receptors coupled to GTP-binding proteins. The G_αS/olf_ is a subunit of the GTP-binding protein, and is expressed only in ciliated neurons (Hansen et al. 2004). The microvillous neurons are shorter in appearance. These olfactory neurons are characterized by having multiple microvilli in the apical domain, hence the name microvillous neurons. They mainly express V1R and V2R receptors and are tuned to detect nucleotides, amino acids (Hansen et al. 2003) and sex pheromones (Zippel et al. 1996). Microvillous neurons express the *G*_αO_, *G*_αq/11_ or *G*_αi3_ subunits of the GTP-binding protein coupled receptors and can be identified immunocytochemically (Hansen et al. 2004). Crypt cells are a less studied cell type. In zebrafish (*Danio rerio*), this type of neuron is characterized by the expression of ORA4 receptors (Oka et al. 2011). Crypt cells are morphologically characterized by their round shape and an invagination with microvilli and cilia at the apical domain. They are also supported by specialized sustentacular cells, characterized by a clear electron-lucent cytoplasm and prominent mitochondria (Hansen and Finger 2000). Crypt cells can be identified by the expression of S-100 and Tropomyosin kinase receptor A (TrkA) (Catania et al. 2003; Germanà et al. 2004). Pear-shaped neurons express mainly Olfactory Marker protein (OMP) and have been shown to detect ATP and related molecules with an adenosine moiety (Wakisaka et al. 2017). Kappe neurons, are characterized by the expression of G_O_ receptors and are similar in shape to crypt neurons, except for the characteristic “cap” structure by which they are named. The odorant specificity of Kappe neurons is still unknown (Ahuja et al. 2014; Klimenkov et al. 2020).

In most fish species, the olfactory system is crucial to a variety of behaviors. These include, foraging, predator/prey interactions (Hamdani and Døving 2007; Tierney et al. 2007), shoaling (Partridge and Pitcher 1980; Kasumyan 2004), homing (Ueda 2019) and reproductive behaviors (Sorensen and Baker 2014). To date, salmonids (Bertmar 1973; Moran et al. 1992), lampreys (VanDenbossche et al. 1995) and cyprinids (Hansen and Zielinski 2005; Pashchenko and Kasumyan 2017) have been the primary fish models used to investigate cellular-level olfactory morphology. Only recently have these methods been applied to non-model species of conservation concern, including elasmobranchs (Ferrando et al. 2007, 2016).

The Delta Smelt, *Hypomesus transpacificus* (Osmeriformes, Osmeridae) is endemic to the Sacramento-San Joaquin Delta and the upper San Francisco Estuary. Since the mid-1980s, their populations have been declining rapidly and dramatically (Teh et al. 2020; CDFW 2021) such that they are now listed as critically endangered by the International Union for Conservation of Nature (IUCN) (NatureServe 2014). Several hypotheses have been proposed to explain this decline and to find ways to recover the population (Sommer et al. 2007). The decision to list Delta Smelt as endangered has led to a plethora of studies geared towards better understanding their biology (Brown et al. 2013; Moyle et al. 2016). However, the olfactory system has been largely ignored even though climate change and anthropogenic alteration of habitats are known to disrupt the sensory behaviors of fish (Tierney et al. 2010; Lürling 2012) including Delta Smelt (Davis et al. 2019). A basic description of the morphology of the olfactory organs of Delta Smelt is needed as a foundation for more applied studies addressing the potential that anthropogenic influences disrupt olfactory function, (Tierney et al. 2010) and contribute to observed declines in population health. Therefore, our aim in this work was to provide a comprehensive anatomical description of the olfactory organ of Delta Smelt, using a combination of histological, ultrastructural, and immunohistochemical approaches.

## Materials and methods

### Animals

Delta Smelt (*Hypomesus transpacificus*) were obtained from the Fish Conservation and Culture Laboratory (FCCL) in Byron, CA, USA. The original stock (F0) came from the San Francisco Estuary and had been bred in captivity for F12 generations using established breeding, genetic, and rearing methods for the species (Fisch et al. 2012; Lindberg et al. 2013).

### Gross morphology

We used 37 (20 males, 17 females) sub-adult and adult Delta Smelt between 263 and 357 Days Post Hatch (DPH; See Table 1). The fish were euthanized following the American Veterinary Medical Association (AVMA) guidelines for the euthanasia of animals (Leary et al. 2013). Briefly, each individual was placed in ice water for 10 min until neither opercular movement nor peduncular reflex were detected. The spinal cord was then cut directly behind the head. After euthanasia, each fish was measured, weighed, and placed in 10% neutral buffered formalin for 48 h.

**Table 1:** Age, life stage, fork length, and weight of Delta Smelt used for Gross Morphology

Age (DPH)	Life stage	Sex		Fork length (cm)	Weight (grams)	Number of individuals
		M	F			
263	Sub-Adult	4	6	6.57 ± 0.70	2.34 ± 0.93	10
294	Sub-Adult	5	2	7.45 ± 0.91	2.92 ± 1.30	7
330	Adult	7	3	7.18 ± 0.68	3.09 ± 0.86	10
357	Adult	4	6	8.26 ± 0.96	5.27 ± 2.52	10

Values are given in mean ± SD

After 48 h of fixation, the olfactory rosettes were dissected at 7 ×–70 × magnification using an Olympus SZH10 (Olympus Corporation, Japan) research stereomicroscope as follows: We introduced 2 µl of Mayer’s Hematoxylin in each nasal cavity to highlight the olfactory rosette. Then, we placed the fixed fish in a plastic tray containing phosphate buffered saline (PBS) and used micro-scissors to remove the skin and surrounding tissue covering the nasal cavity to fully expose the rosette. We recorded the number of lamellae and measured the diameter of each lamellae using AmScope software coupled to an AmScope MU1400 camera (AmScope, Irvine, CA).

## Histology and immunohistochemistry

We used 30 (19 males, 11 females) sub-adult Delta Smelt (mean ± SD, fork length [the length of the fish measured from the tip of the lower mandible to the center of the fork of the tail] of 6.96 ± 0.5 cm and weight of 2.57 ± 0.7 g). The fish were euthanized at 240 DPH, fixed, and dissected as described in the ‘Gross Morphology’ section. Each whole rosette was removed from the nasal cavity, placed in an individual tissue cassette, dehydrated in ascending concentrations of alcohols, cleared in xylene, and embedded in paraffin (TissuePrep™ 2, Fisher Scientific). The tissue blocks were sectioned at 3 µm thickness using a rotatory microtome. Sections were wet mounted on glass slides and stained with Hematoxylin and Eosin (H&E). For immunohistochemical analysis, we used the following primary antibodies (see Table 2 for details: (1) Mouse monoclonal anti *G*_αS/olf_ (C-10, 1:500 dilution sc:377,435 Santa Cruz Biotechnology) against the G protein alpha olf subunit to specifically identify ciliated olfactory neurons, (2) Rabbit polyclonal anti S-100 (1:250 dilution, RB-044-A0 Thermo scientific) to identify crypt neurons, and (3) Rabbit polyclonal anti Calretinin (1:2000 dilution, AB5054 EMD Millipore) to detect the sensory area within the rosette. Additionally, we used anti TRPC2, *G*_αO,_ *G*_αi-3_ and *G*_αq-11_ antibodies (see Table 2) to label crypt cells and microvillous neurons (Hansen et al. 2004; Bettini et al. 2016), however, results were inconclusive and are therefore not reported.

**Table 2:** Primary antibodies used to detect olfactory neurons in the olfactory rosette of Delta Smelt

Target protein	Art number	Lot number	Supplier	Species/sequence
*G*_αS/olf_	SC-377435	C2218	Santa Cruz Biotechnology	Rat 369-394
*G*_αO_	TA333538	QC2517175-90602	ORIGENE	Human
*G*_αO_	SC-13532	F2118	Santa Cruz Biotechnology	N/A
*G*_αi-3_	SC-365422	DO119	Santa Cruz Biotechnology	Rat 339-354
*G*_αq-11_	SC-365906	A2219	Santa Cruz Biotechnology	Human 60-359
TRPC2	LS‑C95010	59673	LSBio	Zebrafish
S-100	RB-044-A0	044A1802J	Thermoscientific	N/A
Calretinin	AB5054-K	3088686	AMD Millipore-Sigma	N/A

All of these markers have been previously used to identify these cell types in fishes and have shown similar staining patterns across several model species, including zebrafish (*Danio rerio*), goldfish (*Carassius auratus*) and catfish (*Ictalurus punctatus*) (Hansen et al. 2003, 2004; Lazzari et al. 2017). The primary antibodies were detected with ImmPRESS (Peroxidase) Polymer Reagent Horse-Anti-Mouse IgG MP-7402 (Vector Labs) for monoclonal antibodies, and ImmPRESS (Peroxidase) Polymer Reagent Horse-Anti-Mouse/Rabbit IgG MP-7500 (Vector Labs) for polyclonal antibodies. The omission of primary antibodies was used as a negative control. The tissue sections were cleared in Xylene substitute (Histoclear), followed by rehydration in descending concentrations of alcohol, and a final rinse in deionized water. The endogenous peroxidase was quenched with 1% hydrogen peroxide in PBS buffer for 20 min, followed by heat antigen retrieval in citrate buffer (Ph 6.1, Target retrieval solution, S1 699, Dako) for 30 min at 92 °C using a commercial vegetable steamer. The nonspecific binding was blocked with 10% normal horse serum (Vector Laboratories, Burlingame, CA, USA), plus 1% bovine serum albumin (Sigma) and 0.1% Tween™ 20 (Fisher BioReagents) for 1 h. The sections were incubated with the primary antibodies for 3 h, then with the secondary antibody for 30 min in a humidified chamber at room temperature. The antigen antibody reaction was revealed with vector Nova-Red peroxidase substrate kit (Vector laboratories, SK-4800) and counterstained with Mayer’s hematoxylin. Images of representative olfactory rosette structures were taken with an Olympus BX60 microscope coupled to a DP71 camera. Pictures were adjusted for brightness and contrast with the CellSenses software (V 1.8.1 Olympus Corporation of the Americas, Center Valley, PA). Image post processing (sizing, intensity, and labeling) was done with Adobe Photoshop (V 20.0.3).

### Ultrastructural morphology

Sub-adult Delta Smelt (four females and one male at 240 DPH) were used for ultrastructural characterization of the olfactory rosette. From each pair of rosettes, one was sampled and prepared for scanning electron microscopy (SEM) and the other for transmission electron microscopy (TEM).

### Scanning electron microscopy (SEM)

Whole olfactory rosettes were fixed in 50% strength Karnovsky’s solution (Fournie et al. 2000) for 48 h and dehydrated in ascending concentrations of ethanol. The rosettes were then critically point dried with CO_2_, mounted in aluminum stubs and double sticky carbon discs, and received three cycles of Gold sputtering. The tissue was examined with a Philips XL 30 scanning electron microscope at 20 keV accelerating voltage. All the procedures were performed in the Biological Electron Microscopy Facility at the University of California, Davis (https://bioem.ucdavis.edu/).

### Transmission electron microscopy (TEM)

Whole olfactory rosettes were fixed in the same way as for SEM, washed in sodium Cacodylate buffer, and post fixed in 1% osmium tetroxide. After osmium fixation (osmification), the tissue was rinsed in 0.1 M sodium cacodylate, dehydrated through a graded ethanol series, transitioned through propylene oxide, and infiltrated and embedded in Eponate-12 epoxy formulation (Eponate-12; Ted Pella Inc., Redding, CA). Thick sections (0.5 µm) were cut, mounted on glass slides, stained with toluidine blue O, and examined by light microscopy. Thin sections (120 nm) were mounted on 300-mesh copper grids, stained with 4% uranyl acetate in 75% ethanol, and post stained in lead citrate. The grids were examined with a FEI Talos L120C transmission electron microscope at 80 keV accelerating voltage (Thermo Fisher Scientific, Hillsboro, OR).

### Statistical analysis

We analyzed the relationships between fork length and discrete variables (age and number of lamellae) using Spearman rank correlations. We analyzed the relationship between fork length and rosette diameter using a Pearson’s product-moment correlation with a Bonferroni correction for multiple comparisons. Results were considered significant at *P* < 0.005. All the analyses and graphs were done with JMP software V 15 (SAS institute, Cary, NC).

## Results

The morphological features of the olfactory rosette of the Delta Smelt resemble what has been observed in other teleosts that have well-developed olfactory systems and behaviors (Kasumyan 2004; Hansen and Zielinski 2005).

### Gross morphology and ultrastructure of the Delta Smelt olfactory rosette

**Fig. 1:**
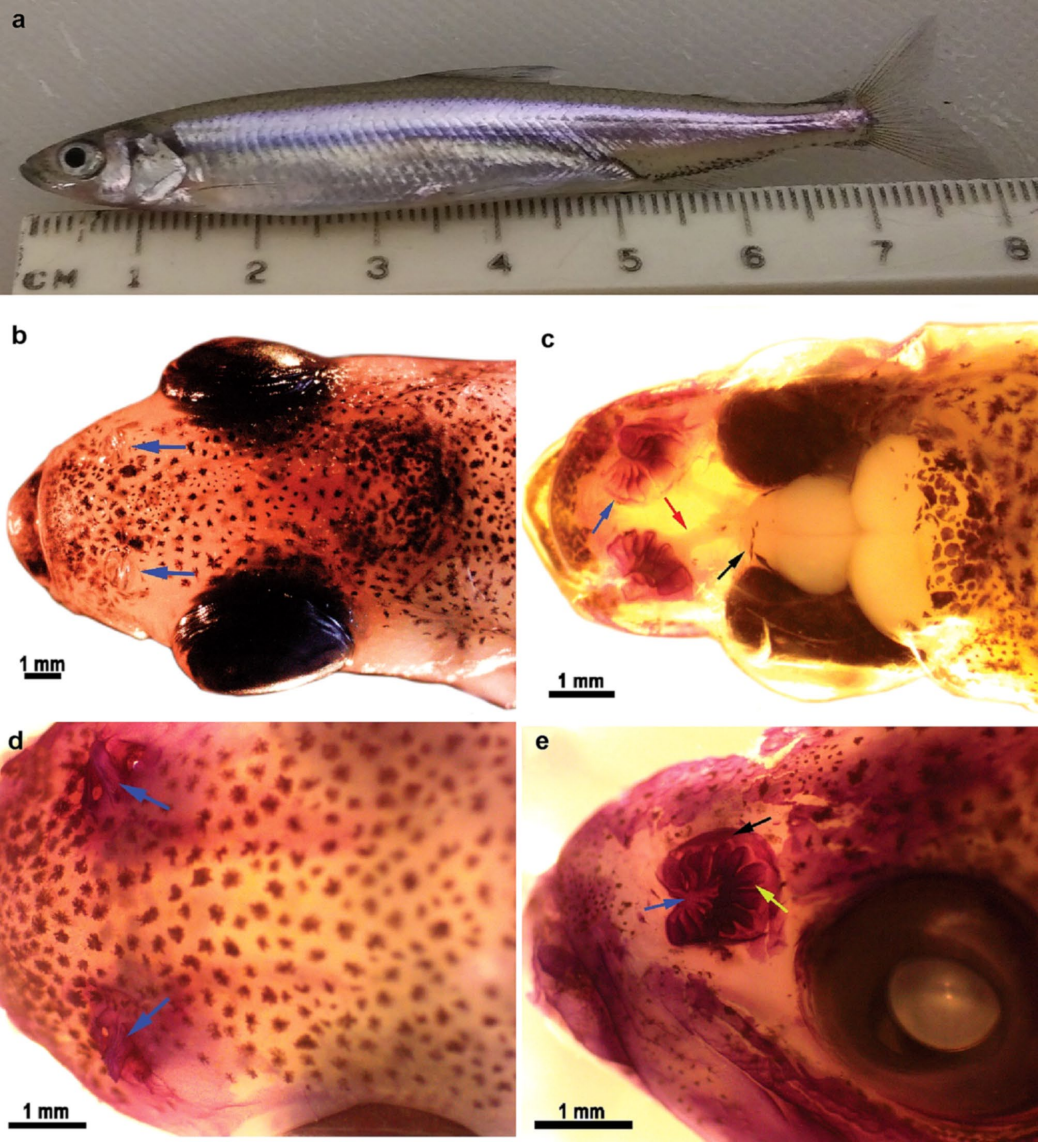
Gross morphology of the Delta Smelt olfactory rosette and main olfactory system structures. **a** Sub adult (294 DPH) Delta Smelt. **b** Nasal cavities (blue arrows) of a 294 DPH Delta Smelt. **c** Dorsal view of the olfactory system in a 294 DPH Delta Smelt. Arrows indicate the olfactory rosette (blue), the olfactory nerves (red) and the olfactory bulbs (black). **d** This photograph was taken with the fish submerged in PBS to highlight the boat sail shaped flap (blue arrows) forming of the anterior and posterior nares. **e** Lateral view of an olfactory rosette in a 263 DPH Delta Smelt. Arrows indicate the central raphe (blue arrow), the lamellae (yellow arrow) and the connective tissue capsule (black arrow)

External examination revealed that Delta Smelt (Fig. 1a) have a round nasal cavity immediately in front of each eye (Fig. 1b). Paired nasal cavities are each covered by a boat sail shaped skin flap that forms anterior and posterior nasal openings for water circulation (Fig. 1d). As in other fish species, the olfactory rosette in Delta Smelt is a round, multi-lamellar sensory structure that sits in the nasal cavity (Fig. 1c, e). The rosette structure is supported and protected by a delicate, fibrous capsule that attaches the lamellar folds to the base and sides of the nasal bones (Fig. 1e).

The lamellae radiate and extend towards the periphery of the nasal cavity, increasing in size from rostral to caudally (Fig. 1e). A central connective tissue raphe (Fig. 1e) provides scaffolding and nutrition to the lamellar epithelium through an abundant network of capillary vessels (see “Fine structure of the Delta Smelt olfactory epithelium”). Within the connective tissue meshwork, the axons of the olfactory neurons coalesce and form nerve bundles which make up the olfactory nerve (Fig. 1c).

**Fig. 2:**
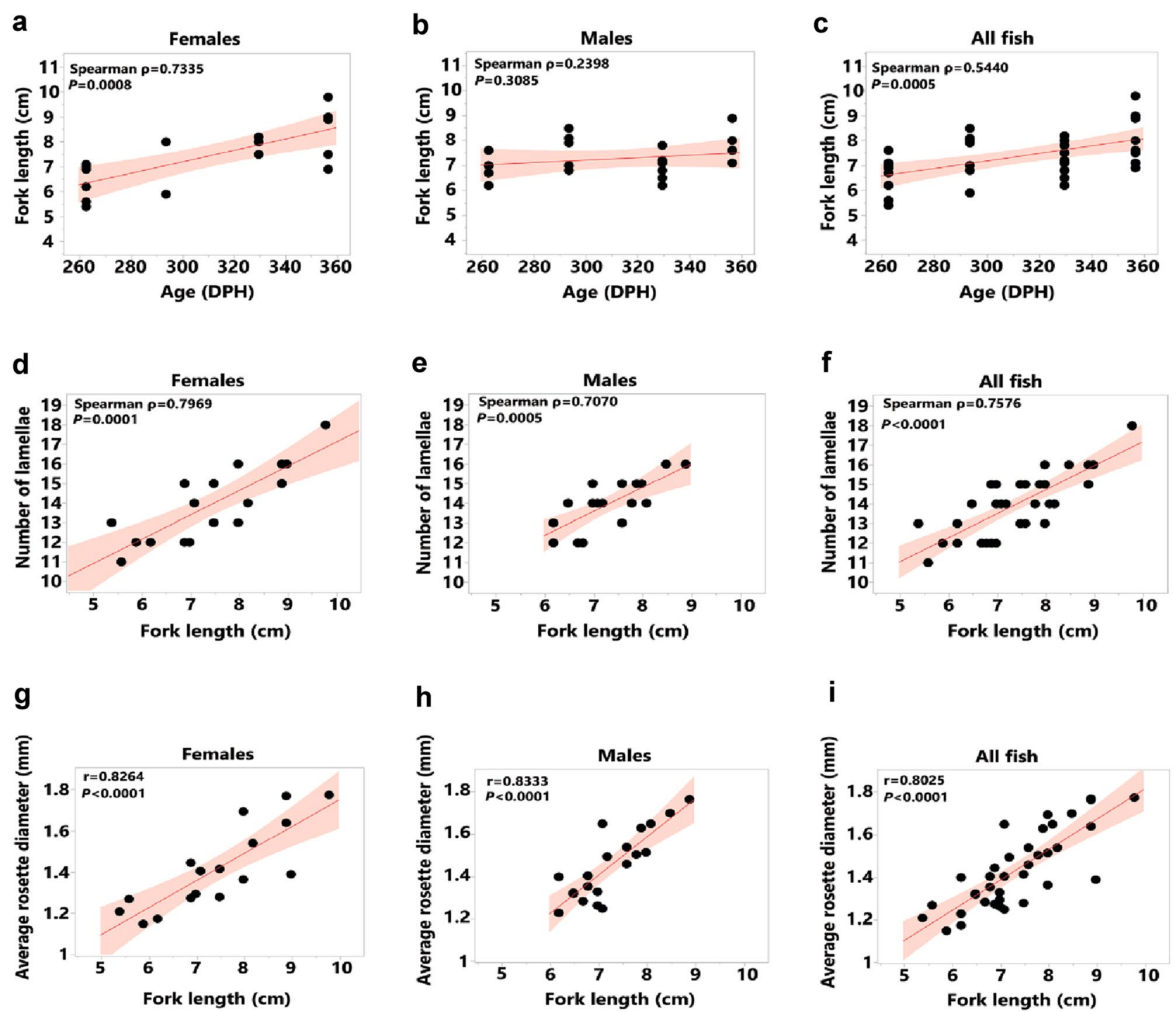
The olfactory rosette of Delta Smelt relative to age and size. The first row **a**–**c** illustrate the relationship between age and fork length for females **a**, males **b**, and males and females considered together **c**. The second row **d-f** illustrates the relationship between fork length and number of lamellae of one rosette for females **d**, males **e** and both males and females considered together **f**. The bottom row illustrates the relationship between fork length and average rosette diameter (*n* = 2 rosettes per fish) for females **g**, males **h** and both males and females considered together **i**. In panels **a**–**f**, *P* values were determined using Spearman’s rank correlation, and in panels **g**–**i,** *P* values were determined using a Pearson’s product-moment correlation. For all data, Bonferroni correction for multiple comparison (*P* = 0.005) was applied (see “Methods”)

We found that the number of lamellae and diameter of the rosette varied with age and fork length (Table 3; Fig. 2**)**. In general, smaller, or younger fish had smaller rosettes and fewer lamellae than larger or older fish. We also found that fork length increased with age in females (Spearman’s rank correlation, *Ρ* = 0.7335, *P* = 0.0008), but not males (Spearman’s rank correlation, *Ρ* = 0.2398, *P* = 0.3085) (Fig. 2a, c). However, in both sexes, the average diameter of the rosette and the number of lamellae increased with fork length (Spearman’s rank correlation, number of lamellae vs fork length, females: *Ρ* = 0.7969, *P* = 0.0001, males: *Ρ* = 0.7070, *P* = 0.0005; Pearson’s product-moment correlation, average rosette diameter vs fork length, females: *r* = 0.8264, *P* < 0.0001; males: *r* = 0.8333, *P* < 0.0001) (Fig. 2d, i), suggesting that the overall size of the peripheral olfactory system scales with size rather than age.

**Table 3:** Size of the olfactory rosette of Delta Smelt relative to sex, days post hatch (DPH), fork length and weight

Sex	Age (DPH)	Number of Individuals	Number of Lamellae	Rosette Diameter (mm)	Fork length (cm)	Weight (g)
Females	263	6	11–14	1.27 ± 0.09	6.36 ± 0.74	2.05 ± 0.81
	294	2	12–13	1.42 ± 0.31	6.95 ± 1.48	2.18 ± 1.83
	330	3	13–16	1.39 ± 0.12	7.90 ± 0.36	3.69 ± 0.38
	357	6	15–18	1.57 ± 0.17	8.50 ± 1.07	6.12 ± 2.87
Males	263	4	12–14	1.31 ± 0.09	6.87 ± 0.58	2.77 ± 1.04
	294	5	12–16	1.54 ± 0.16	7.66 ± 0.73	3.22 ± 1.16
	330	7	12–14	1.37 ± 0.10	6.87 ± 0.54	2.84 ± 0.90
	357	4	14–16	1.62 ± 0.11	7.90 ± 0.76	4.00 ± 1.32

Values are given in mean ± SD, one rosette was measured (for number of lamellae) for each fish

**Fig. 3:**
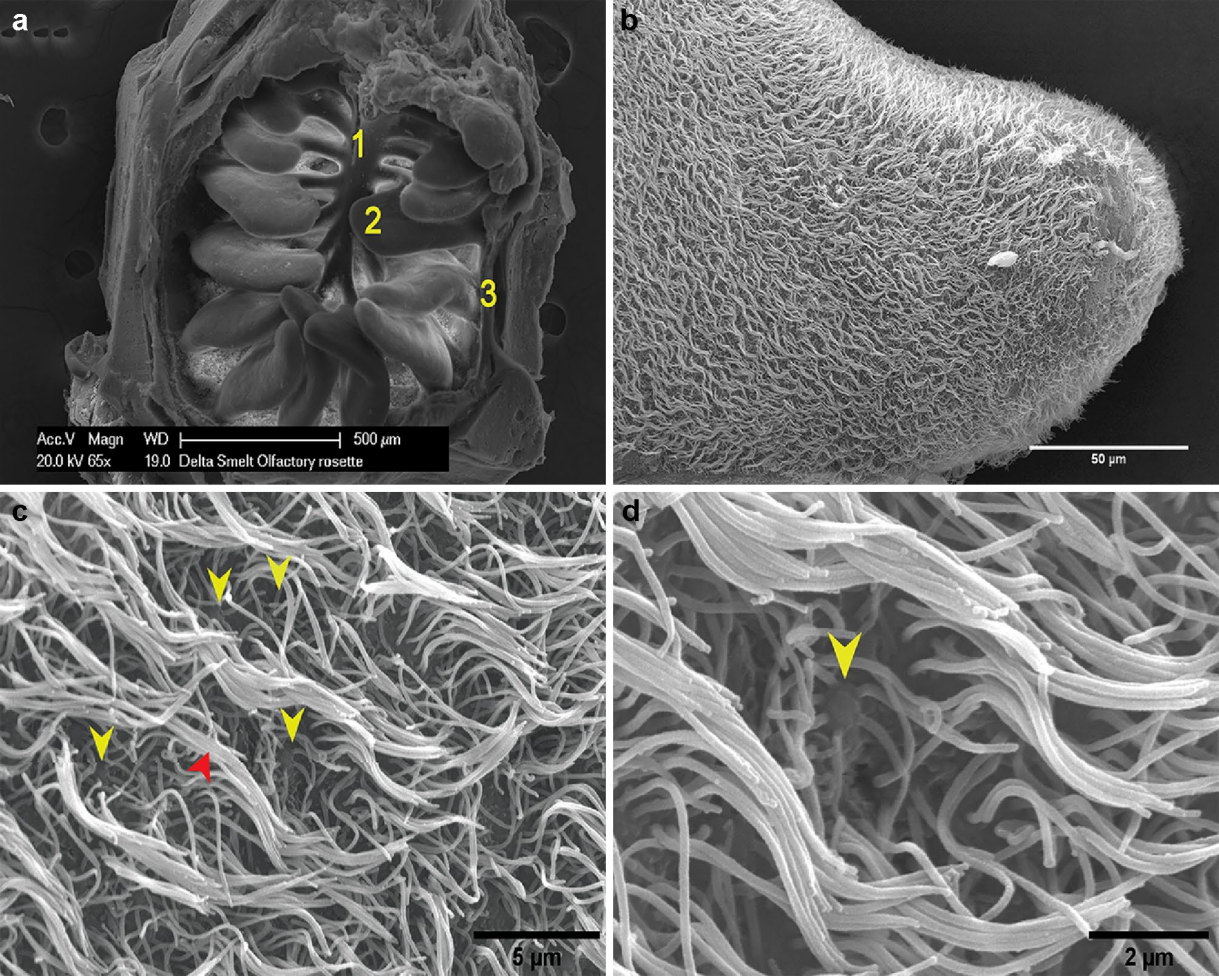
Ultrastructural features of the surface epithelium of a Delta Smelt olfactory rosette. **a** Scanning electron micrograph of an olfactory rosette showing the lamellar array surrounded by the fibrous capsule (1 = central raphe, 2 = olfactory lamella, 3 = capsule, 65X, SEM). **b** Higher magnification view of the surface of a lamella, showing the dense mat of ciliary structures covering the whole lamellar surface, × 650, SEM. **c** The olfactory knobs of several olfactory neurons (yellow arrowheads) embedded in the dense ciliary mat of the lamellar surface are shown. The abundant upright cilia (red arrowhead) likely project from non-sensory sustentacular ciliated cells, × 5000, SEM. **d** Note the sensory cilia projecting from the olfactory knob (yellow arrowhead) of the sensory neuron, × 12000, SEM. For all panels, the subject was a 240 DPH Female Delta Smelt

Under scanning electron microscopy (SEM), we found the olfactory lamellae lacked secondary folds or sensory islands that have been reported in other fish species (Fig. 3a) (Thommesen 1983; Theisen et al. 1991). Instead, each lamella was completely covered on each side by a thick mat of cilia (Fig. 3b). Under SEM, the ciliated olfactory neurons were identified by the presence of olfactory knobs (Fig. 3c). These structures were found to protrude, forming a raised membrane cap characteristic of ciliated olfactory neurons (Fig. 3d**)**. We were not able to conclusively identify microvillar neurons by SEM due to the thick mat of cilia covering the lamellar surface.

### Fine structure of the Delta Smelt olfactory epithelium

**Fig. 4:**
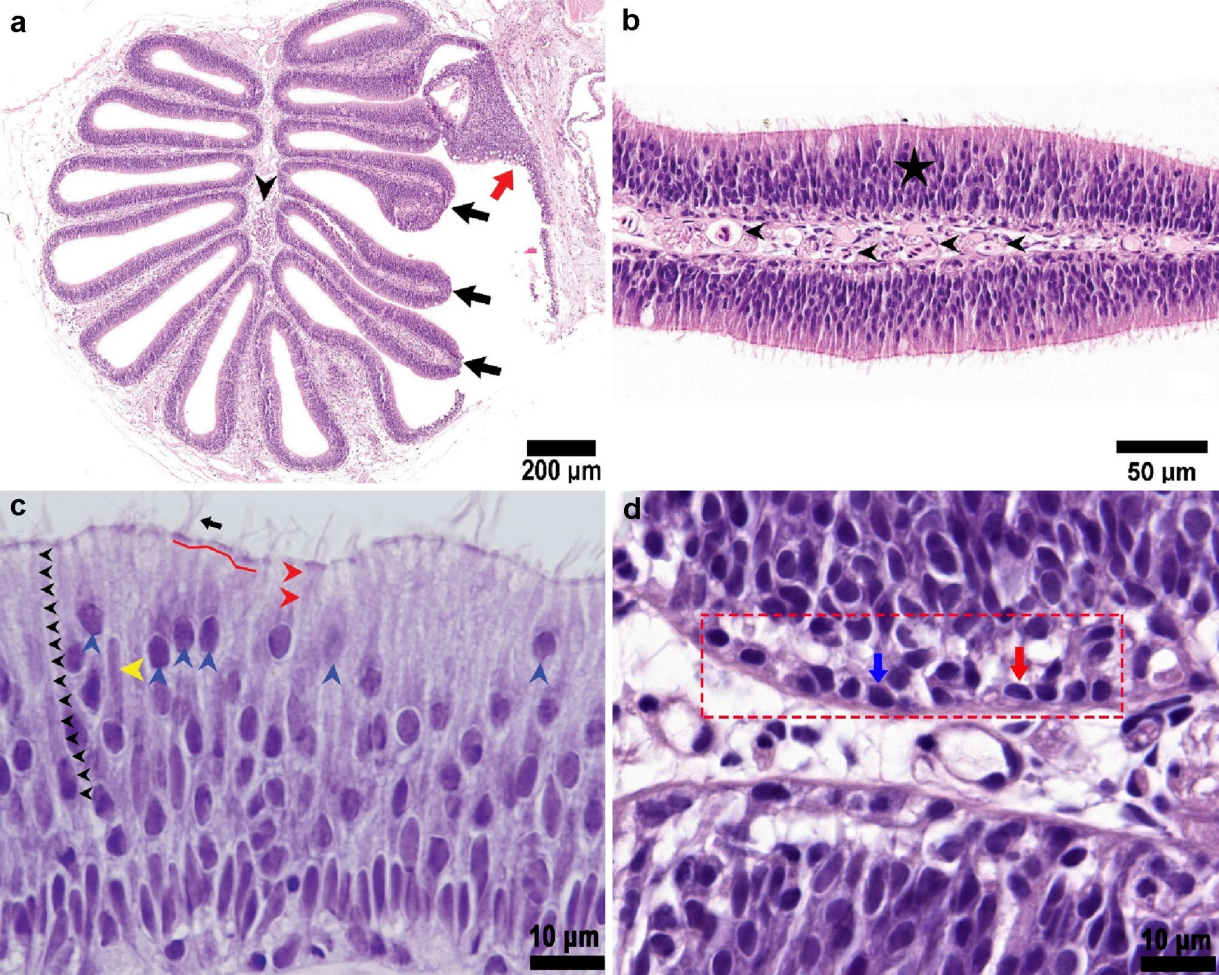
Olfactory rosette and olfactory epithelium in the Delta Smelt. **a** Horizontal section of the whole olfactory rosette showing multiple lamellae (black arrows), that contain the sensory epithelium supported by a delicate fibrovascular stroma (lamina propria, arrowhead). The rosette capsule (red arrow, Fig. 11) surrounds the whole organ, × 40, H&E stain. **b** Single olfactory lamella covered by olfactory epithelium (black star), supported by a fibrovascular stroma with abundant capillary vessels (black arrowheads), × 400, H&E stain. **c** Detail of the olfactory epithelium showing a heterogeneous cell population. Some cells have a long body and a round to ovoid nucleus near the base. A representative cell is highlighted by black arrowheads. Other cells (blue arrowheads) have round, more superficial nuclei, and shorter cell bodies. These cells likely represent a different morphotype of bipolar sensory neurons. Sustentacular cells (red arrowheads) tend to have an elongated nucleus (yellow arrowhead), and a wider apical surface than ciliated sensory neurons. A red line highlights the ciliary basal bodies at the epithelial apical surface from which several cilia project to the surface (black arrow) × 1000, H&E stain. **d** Basal domain of the olfactory epithelium (red squared area), showing two main basal cell types. The horizontal basal cells (red arrow) characterized by an elongated nucleus, and the globose basal cells (blue arrow), characterized by a round nucleus, × 1000, H&E stain

**Fig. 5:**
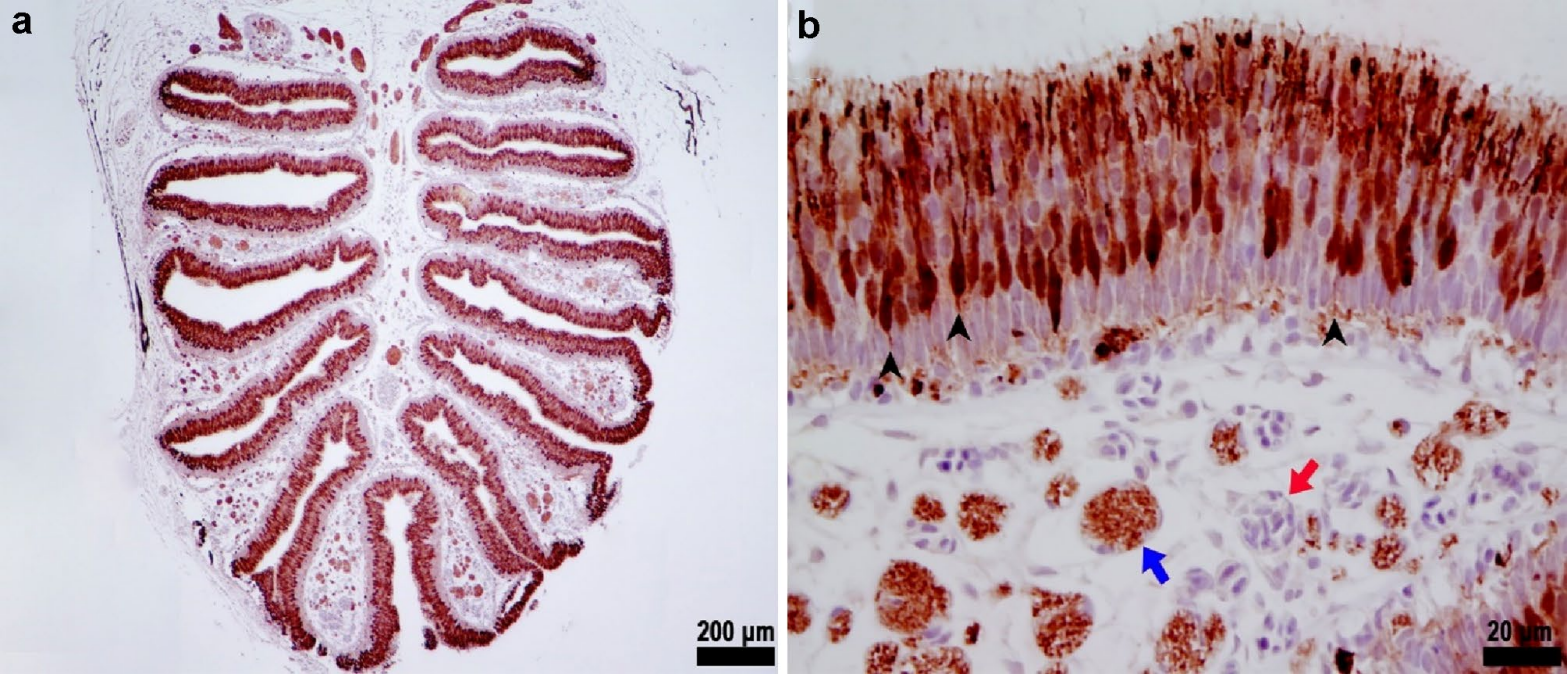
Calretinin immunolabeling highlighting the distribution of sensory neurons in the olfactory lamellae of Delta Smelt. The Calretinin immunohistochemical labeling (red staining) indicates a heterogeneous population of sensory neurons in the olfactory epithelium. **a** Whole olfactory rosette showing the distribution of sensory neurons, × 40, Light microscopy photomicrograph, Immunohistochemical stain for Calretinin. **b** Higher magnification of sensory neurons. Axons (black arrowheads) can be seen projecting from olfactory neurons towards the lamina propria. Nerve bundles (blue arrow) and capillary vessels (red arrow) are abundant in the lamina propria, × 400, Light microscopy photomicrograph, Immunohistochemical stain for Calretinin

**Fig. 6:**
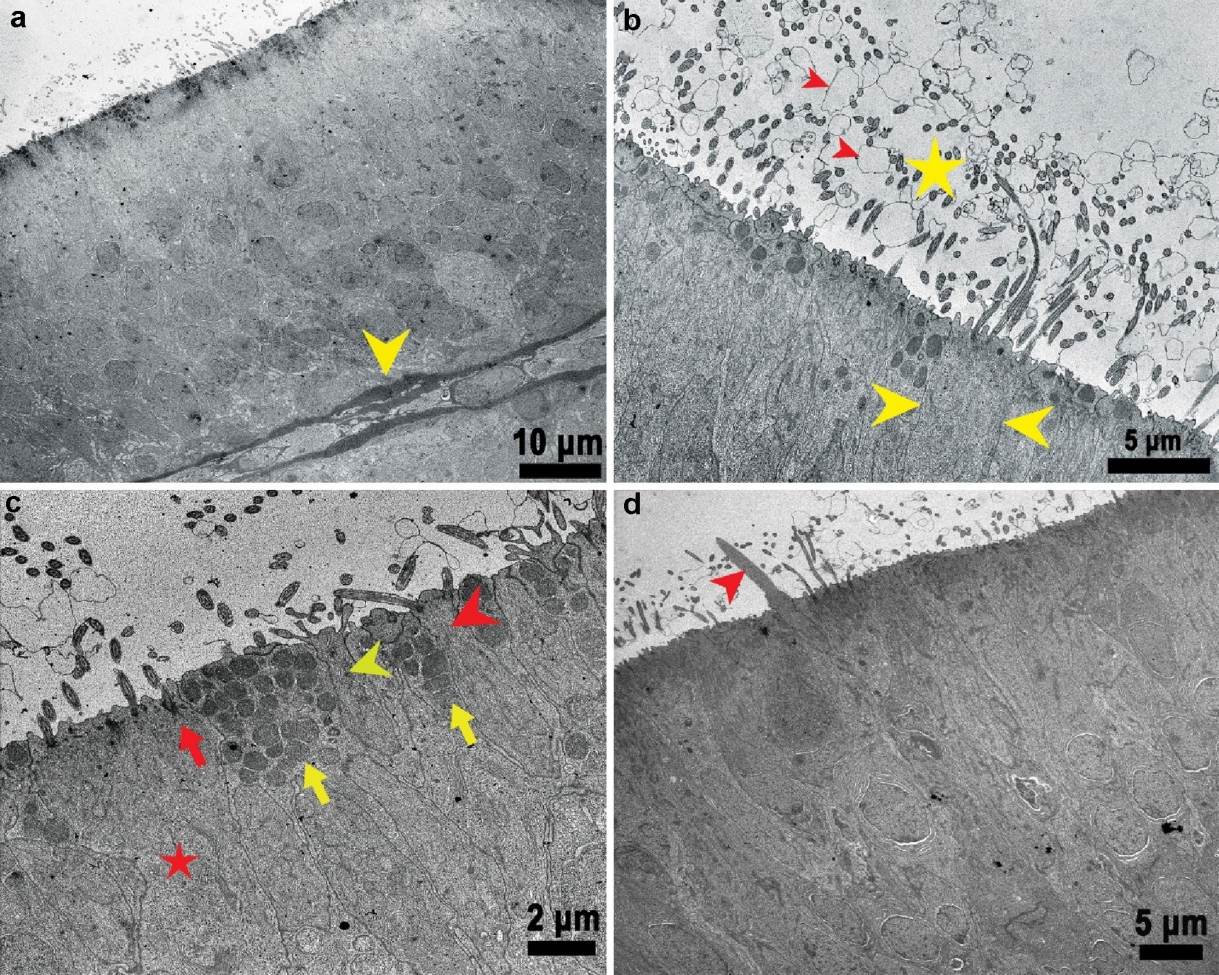
Diversity of apical modifications in the olfactory epithelium of Delta Smelt. **a** Transmission Electron micrograph from the basal lamina to the surface of the olfactory epithelium. The arrowhead (yellow) shows the basal lamina in which the epithelium is settled, × 600, TEM. **b** Detail of the epithelial surface. Note the epithelium is covered by cilia (yellow star) and electron dense filamentous material (red arrowheads indicate two examples). Cilia are known to project from sensory neurons (See also Fig. 7d) and non-sensory ciliated sustentacular cells (yellow arrowheads), × 1500, TEM. **c** Detail view of the epithelial surface showing a diversity of apical modifications of cell types. Ciliated sustentacular cell (red star) with prominent microtubular basal bodies and rootlets (red arrow). Secretory sustentacular cells with abundant electron dense secretory granules are also indicated (yellow arrows). The microvillous neurons (yellow arrowhead) and ciliated sensory neurons (red arrowhead), are interspersed between the sustentacular cells, × 2500, TEM. **d** Detail of a rod-shaped apical modification (red arrowhead) that was observed in some cells, × 1250, TEM

We next examined the fine structure of the olfactory epithelium using light and transmission electron microscopy (TEM). We found that the whole surface of the olfactory lamellae of Delta Smelt was composed of a sensory epithelium supported by a delicate fibrovascular stroma, with abundant nerve bundles and blood vessels (Figs. 4, 5). The epithelium rested in a delicate thin basal lamina that separated it from connective tissue in the central raphe (Fig. 6a). The olfactory epithelium itself was a pseudo-stratified columnar layer of 43.86 ± 10.67 μm (mean ± SD) thick composed of a heterogeneous cell population including bipolar neurons, sustentacular, basal (Fig. 4c, d), and goblet cells (Fig. 8b). The epithelial surface was mostly composed of upright cilia from sustentacular cells, which covered the sensory cilia and microvilli from ciliated and microvillous sensory neurons (Fig. 6b, c). A prominent apical basophilic band consisting of the rootlets and basal bodies of cilia from ciliated sustentacular cells typically spanned the entire epithelial surface (Figs. 4c, 6c).

### Neuronal diversity of the olfactory epithelium of the Delta Smelt

Using a variety of immunohistochemical markers (Table 4), we were able to identify ciliated OSNs, microvillous neurons and crypt cells. These cell types appeared to be distributed in a heterogenous population throughout the epithelium. The various cell types are described below.

**Table 4:** Summary of immunocytochemical characteristics of Delta Smelt olfactory neurons

Target protein	Immunostaining distribution		
	Ciliated OSNs	Microvillous neurons	Crypt neurons
*G*_αs/olf_	Cytoplasm, olfactory knob, sensory cilia, olfactory nerve bundles *(fila olfactoria*)	None	None
Calretinin	Cytoplasm and nucleus	Cytoplasm and nucleus	Cytoplasm and nucleus
S-100	Cytoplasm and nucleus	Cytoplasm and nucleus	Cytoplasm and nucleus

**Fig. 7:**
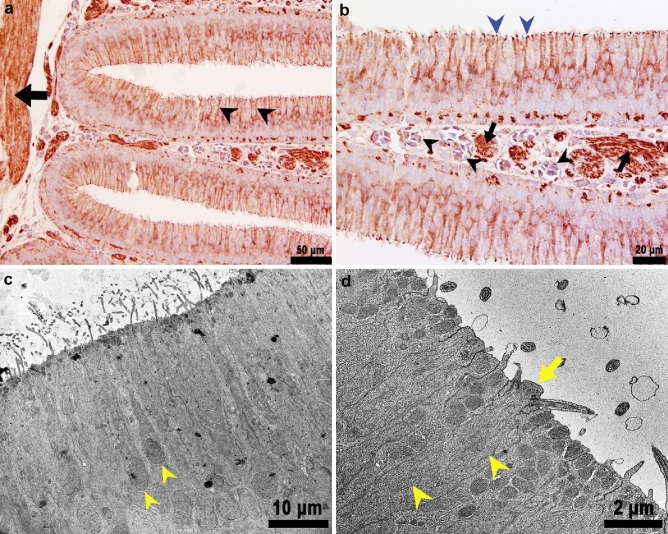
Immunohistochemical and ultrastructural characteristics of ciliated olfactory sensory neurons (ciliated OSNs). **a** Ciliated OSNs neurons stained with anti *G*_αs/olf_ antibodies, highlighting their long, slender cell body and dendrite (black arrowheads). The prominent *fila olfactoria* in the lamina propria labeled with anti *G*_αs/olf_ antibodies is also shown (black arrow), × 200, *G*_αs/olf_ Immunohistochemistry, bright field microscopy. **b** Higher magnification of (**a**). The olfactory knobs are well defined (blue arrowheads) forming a linear staining pattern on the epithelial surface. The *lamina propria* contains abundant blood vessels (black arrowheads) and nerve bundles (black arrows) in cross section, × 400, *G*_αs/olf_ Immunohistochemistry, bright field microscopy. **c** Transmission electron microscopy of ciliated OSNs. These cells project a long, slender dendrite towards the epithelial surface (yellow arrowheads), × 800, TEM. **d** The dendrites end with an olfactory knob (yellow arrow). Multiple tubular mitochondria are observed in the dendrite process (yellow arrowheads), × 4000, TEM

*Ciliated olfactory sensory neurons *(*ciliated OSNs*): The cell bodies of ciliated OSNs were elongated and irregular and were usually found in the mid and lower depths of the epithelium near the basal membrane. Ciliated OSNs were characterized by a round to oval nucleus that occupied most of the neuronal soma, and by a long and slender dendrite extending to the epithelial surface (Fig. 7). Ultrastructurally, we were also able to identify ciliated OSNs by their characteristic olfactory knob, and five to six sensory cilia 2–3 µm in length projecting from it (Fig. 7d). The cell bodies and dendrites had abundant tubular mitochondria (Fig. 7d). Their nuclei were typically round to oval and were observed in the medial to basal level of the sensory epithelium. The nuclei contained abundant electron lucent agranular euchromatin. We also observed small clumps of electron dense, granular heterochromatin in the nuclear periphery (Fig. 7c).

Delta Smelt ciliated OSNs were positive for *G*_αs/olf_, Calretinin and S-100 (Figs. 5, 7, 9). The *G*_αs/olf_ expression was confined to ciliate olfactory neurons and showed a coarse granular cytoplasmic and dendritic staining pattern. The olfactory knobs and cilia were densely stained for *G*_αs/olf_ and formed a rim on the surface of the epithelium (Fig. 7b). Calretinin (Fig. 5) and S-100 (Fig. 9 and Online resource Fig. 1) were distributed within the nucleus and the cytoplasm, all along the dendritic process to the olfactory knob and olfactory cilia on the epithelial surface. We occasionally observed the axons of ciliated sensory neurons stained for calretinin and S-100. Individual axons could be visualized projecting to the basal layer and forming nerve bundles (*fila olfactoria*) in the lamina propria, which strongly stained with calretinin, *G*_αs/olf_ (Figs. 5b, 7a, b) and S-100 (Online resource Fig. 1).

**Fig. 8:**
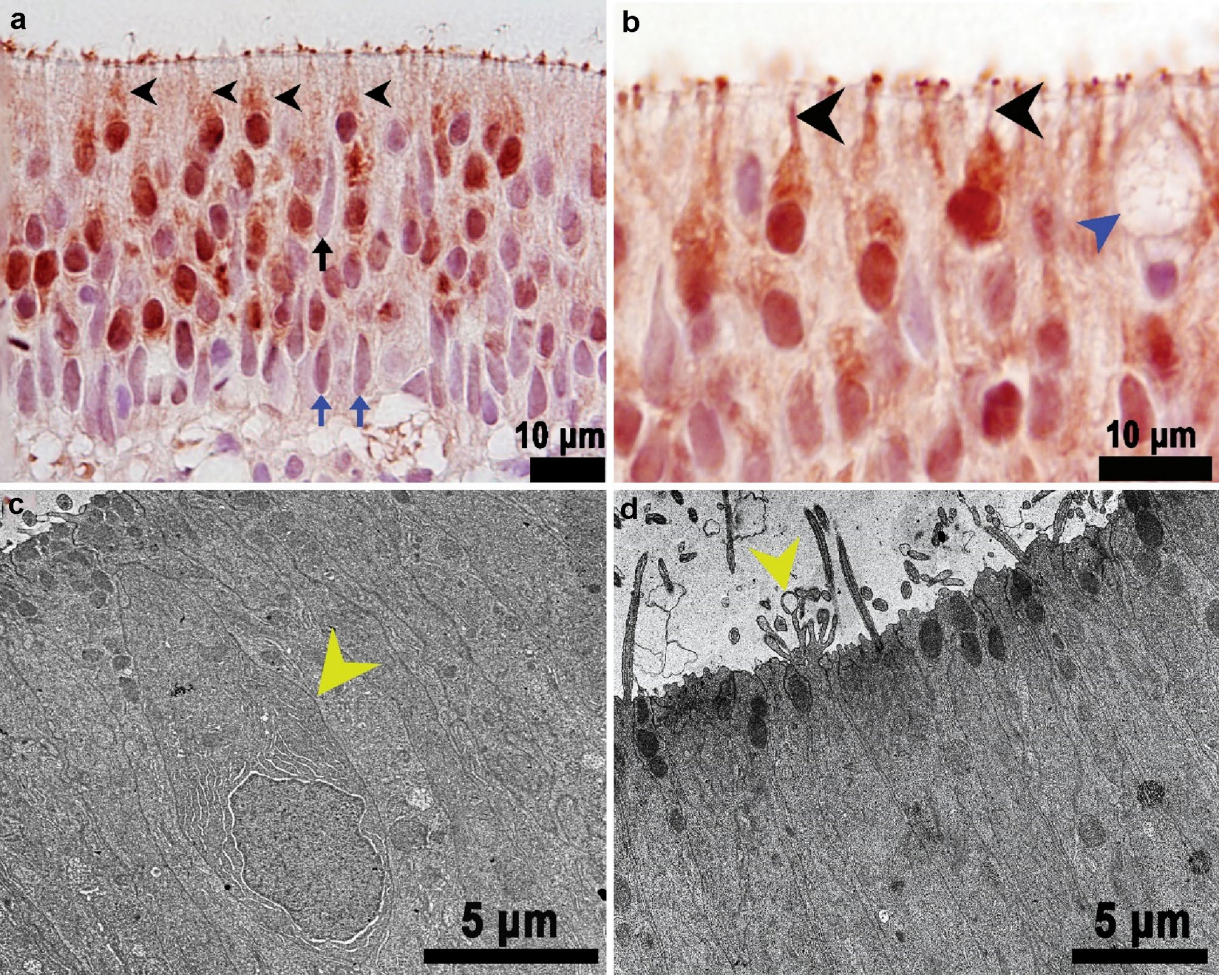
Cross section of a lamella showing the microvillous neurons in the olfactory epithelium of Delta Smelt. **a** Microvillous neurons are positive for S-100 (black arrowheads). Note the short, pear-shaped body with a round to ovoid nucleus. The blue arrows point to the nucleus of sustentacular cells which are S-100 negative and intermediate-basally located, × 1000, S-100 Immunohistochemistry, bright field microscopy. The black arrow points to the nucleus of a sustentacular cell in the mid-apical region. **b** Closer view of microvillous neurons. The image shows microvillous neurons near the surface of the olfactory epithelium with occasional, short dendrites (black arrowheads), the blue arrowhead points to a goblet cell, × 1000, S-100 Immunohistochemistry, bright field microscopy. **c** TEM photomicrograph near the epithelial surface showing the body of a microvillous neuron (yellow arrowhead) characterized by an electron dense cytoplasm with few mitochondria × 1200, TEM. **d** TEM photomicrograph of a microvillous neuron projections. Several short microvillous extensions are illustrated projecting from a microvillous neuron to the epithelial surface (yellow arrowhead), × 2000, TEM

*Microvillous Neurons*: Microvillous neurons were characterized by a short pear-shaped soma, a short dendrite, and a round, superficial nucleus (Fig. 8a). These neurons were easily distinguished from other types of neurons by a dendritic process that was short and thin (Fig. 8b) and the presence of microvilli on the apical domain. The cytoplasm of microvillous cells was typically electron dense with few mitochondria. The nuclei tended to be round, euchromatic and lack significant peripheral heterochromatin (Fig. 8c). The microvilli appeared to be thinner than the sensory cilia of ciliated OSNs (Fig. 8d). The S-100 strongly stained nuclei, cytoplasm, and microvilli (Fig. 8a, b), however, immunostaining for calretinin was weaker and more variable in microvillous neurons. In addition, the nuclei tended to be stained more weakly, with clearer cytoplasm (data not shown).

**Fig. 9:**
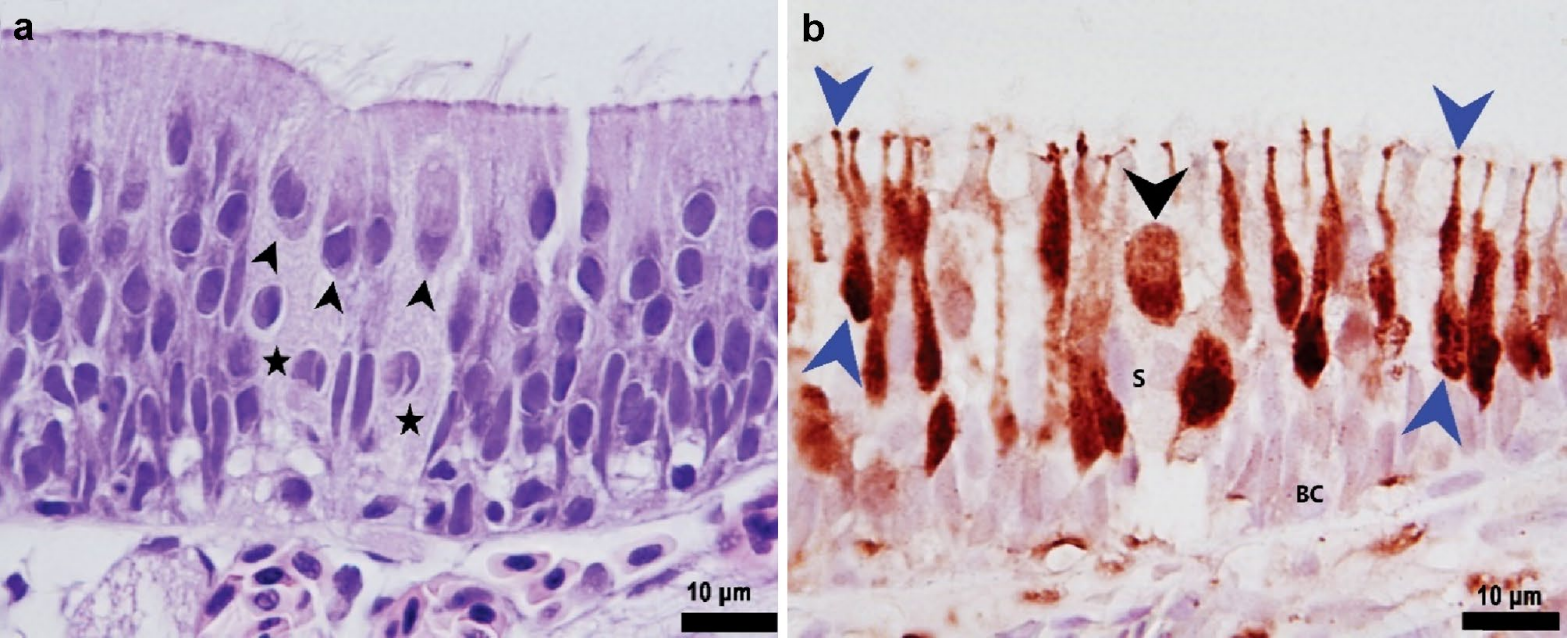
Histological features of the crypt neurons in an adult Female Delta Smelt. **a** Cluster of crypt cells (black arrowheads) surrounded by several supporting cells (black stars), × 1000, H&E Stain. **b** S-100 immunostaining identifying the crypt neurons (black arrowhead) and ciliated OSNs (blue arrowheads) in the olfactory epithelium, basal cells (BC) and sustentacular cells (S) were not stained, S-100 Immunohistochemistry, × 1000. Female Delta Smelt, 240 DPH

*Crypt Neurons*: Crypt neurons are ovoid neurons that were found to occur in clusters of three to four cells surrounded by well-developed sustentacular cells (Fig. 9a). Immunohistochemically, crypt cells were easily identified by this distinctive shape and the expression of calretinin and S-100 in the cytoplasm and nuclei (Fig. 9b). Crypt neurons stained weakly for calretinin, although the staining was denser in the nucleus than in the cytoplasm. The apical domain also occasionally stained, highlighting small microvilli (data not shown).

### Non-neuronal cell populations in the olfactory epithelium of the Delta Smelt

*Sustentacular cells:* Sustentacular cells surrounded olfactory neurons and formed cytoplasmic-membrane folds resembling cytoplasmic invaginations (Online resource Fig. 2a). We observed both ciliated and non-ciliated sustentacular cells. These cell types were characterized by an elongated cytoplasm and a sausage-shaped nucleus located primarily in the basal domain of the epithelium (Fig. 8a). Occasionally, nuclei were also observed in a more intermediate position (see Fig. 8a, black arrow). The nucleus had an electron-lucent appearance with abundant euchromatin (Online resource Fig. 2a). The ciliated non-sensory sustentacular cells were characterized by a prominent ciliated apical domain with a ciliary apparatus (see Fig. 6b, c). The cilia were observed to be anchored to the cell by prominent basal bodies that formed the rootlets of the cilia (Fig. 6c). The apical portion of these cells contained abundant mitochondria and small, electron dense granules (presumably glycogen) interspersed close to the mitochondria (data not shown). The upright cilia were observed to have a 9 + 2 microtubular array, and an average length of 11.56 ± 2.69 (mean ± SD) µm, measured from the epithelial surface. We also observed electron dense filamentous material attached to the ciliary surface (Fig. 6b, fine circular structures). The non-ciliated sustentacular cells contained prominent electron dense secretory granules in the apical domain, suggesting they functioned as secretory cells (Fig. 6c). The secretory granules were round to ovoid, with an average diameter of 0.57 ± 0.09 (mean ± SD) µm. These granules had a thin membrane that enveloped the granular contents. The granular contents were occasionally arranged in stripes of electron dense material that gave the granule a lamellated/striped appearance. We also observed several clusters of neurons surrounded by prominent sustentacular cells visible in some areas of the olfactory lamellae, mainly in the folds between contiguous lamellae (Online resource Fig. 2). These sustentacular cells were characterized by an abundance of mitochondria and smooth endoplasmic reticula. The cytoplasm of sustentacular cells was electron lucent when compared to that of sensory neurons. This was mainly due to rich Golgi cisternae and smooth endoplasmic reticula. These sustentacular cells had small microvilli in the apical domain, but they did not have cilia (Fig. 9a, Online resource Fig. 2).

In general, the nuclei and cytoplasm of sustentacular cells were not labeled by any of the markers used to identify sensory neurons (S-100, Calretinin, *G*_αS/olf_); nor were the cilia on the surface of the epithelium labeled (Figs. 8a, 9b).

*Basal cells*: The epithelial basal layer consisted of two identifiable cell types. One type of basal cell could be distinguished as having a round, globoid shape with a round, highly basophilic nucleus, whereas the second type had an elongated shape with ovoid nucleus parallel to the basal lamina (Fig. 4d). Occasionally we observed cells that had elongated and intensely basophilic nuclei, closer to the epithelial surface in the middle and apical portions of the epithelium. Presumably, these were basal cells in the process of developing into mature receptor cells, however the basal cells were not positive for any of the markers used to identify sensory neurons.

### Other cellular populations

**Fig. 10:**
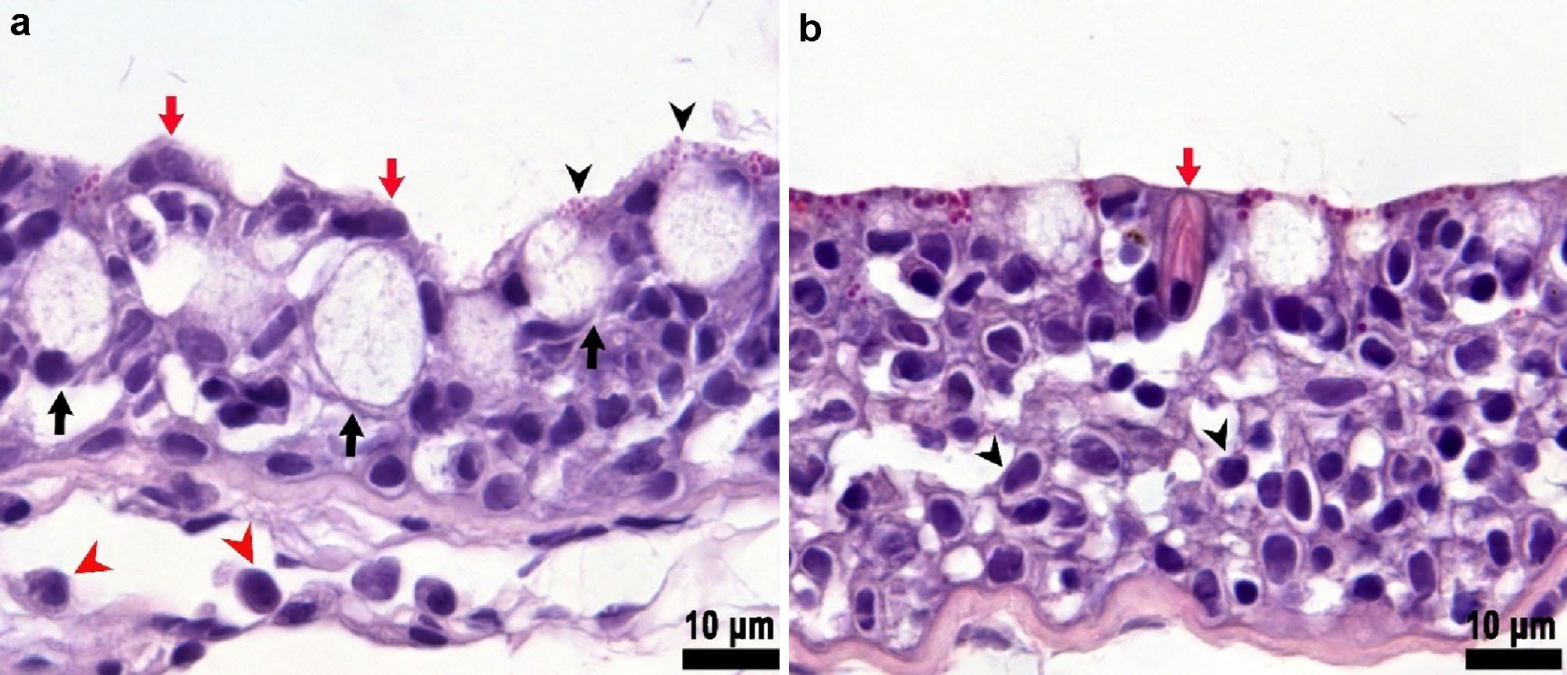
Histological features of the olfactory rosette capsule epithelium. **a** The capsular epithelium (See Fig. 4a, for sub-gross morphology) is composed by several cell types, including pavement cells (red arrows), abundant goblet cells (black arrows), and immune cells (red arrowheads). Abundant bright eosinophilic granules are observed in the epithelial surface (black arrowheads), × 1000, H&E Stain. **b** Abundant resident immune cells infiltrate the capsular epithelium. These cells are characterized by a round to elongated basophilic nuclei (arrowheads). Also, occasionally rodlet cells are observed residing within the epithelium (red arrow), × 1000, H&E Stain

Normally, a few mucous-secreting goblet cells were scattered within the olfactory epithelium (Fig. 8b), and they were more abundant and prominent on the surface of the capsule in which the rosette sits (Fig. 10a). The capsule epithelium showed eosinophilic bright refringent granules interspersed within the epithelial cells on the surface (Fig. 10a). Most of the resident immune cells were found within the capsular connective tissue, and were characterized by round, highly basophilic central nuclei, and small amounts of cytoplasm. Occasionally, rodlet cells were also seen in the capsular epithelium. These cells were characterized by having an ovoid, elongated, brightly eosinophilic cytoplasm containing several birefringent rod structures (Fig. 10b).

Finally, using transmission electron microscopy, we observed cells with a different apical modification consisting of a thick knob with a single prominent rod-like structure protruding to the surface. We did not observe ciliary structures, microtubules, or centromeres on this cell type. This rod structure resembled a dendrite that was thicker than a normal cilium (Fig. 6d).

## Discussion

Our morphological examination suggests that the Delta Smelt has a well-developed peripheral olfactory system, that is in many ways similar to other highly olfactory fish species that have previously been described (Hansen et al. , 2003, 2004; Hansen and Zielinski 2005). The overall implication is that these fish have a functional and potentially keen sense of smell that needs to be further explored.

### Morphology of the olfactory rosette

The Delta Smelt have a pair of well-developed olfactory rosettes, typical of the morphology of macrosmatic fish (Kasumyan 2004). Within each rosette, lamellae are arranged in a radial array, with lamellae increasing in size caudally. The number of lamellae and lamellar arrangement are similar to other fish species, including zebrafish (*Danio rerio*) (Hansen and Eckart 1998), goldfish (*Carassius auratus*) (Hansen et al. 2004), and chum salmon (*Oncorhynchus keta*) (Kudo et al. 2009). As with other species (Kudo et al. 2009; Pashchenko and Kasumyan 2017), we found that additional lamellae were added as fish grew, suggesting that olfactory function is adaptive from juvenile life stages into maturity (Hara and Zielinski 1989; Schluessel et al. 2008; Kudo et al. 2009; Pashchenko and Kasumyan 2017).

As in other fishes, most of the olfactory neurons are in the lateral parts of lamellae and embedded within a mat of cilia that are thought to aid in the sampling and movement of odorants within the sensory surfaces of the olfactory rosette (Pashchenko and Kasumyan 2017). Ciliated sustentacular cells, in particular, have been shown to be involved in driving microcurrents over the olfactory lamellae of other fish species (Reiten et al. 2017; Cox ). Together with the nasal flap, ciliary movement is presumed to generate unidirectional water currents through the nares. In our gross anatomical study, we did not find evidence of accessory sacs that might help in the movement of water into the nares, suggesting that ventilation occurs by swimming activity or opercular movement linked to gill ventilation. While accessory sacs have been described in some fish, they are more commonly present in bottom dwelling or less active fish and are thought to be an adaptation to draw water into the nasal cavity in the absence of a current (Burne 1909; Døving et al. 1977; Nevitt 1991).

We found that Delta Smelt are unusual in that both sides of the olfactory lamellae are completely covered with sensory epithelium interspersed with non-sensory epithelium. This is not the case for goldfish (*Carassius auratus*), (Hansen et al. 2004), zebrafish (*Danio rerio*) (Hansen and Eckart 1998), salmonids (Thommesen 1983), guppies (*Poecillia reticulata*) (Lazzari et al. 2007) or catfish (*Ictalurus punctatus*) (Caprio and Raderman-Little 1978; Theisen et al. 1991), which have well-differentiated regions or ‘islands’ of sensory and non-sensory epithelium in the olfactory lamellae. This may reflect fewer infoldings in the lamellae of Delta Smelt.

### Cytology of sensory neurons

The neuronal populations in the olfactory epithelium were similar to those of other Teleosts and showed similar morphological and immunohistochemical features as well (Hansen and Zielinski 2005). Ciliated OSNs from Delta Smelt had a long dendrite and ciliated olfactory knob, and both the number and length of cilia were similar to those reported in zebrafish (*Danio rerio*) (Hansen and Eckart 1998), brown trout (*Salmo trutta*) (Moran et al. 1992) and goldfish (*Carassius auratus*). The open and agranular euchromatin in these neurons reflected their high transcriptional activity (Kierszenbaum and Tres 2019). Interestingly, in Delta Smelt ciliated OSNs, we observed *G*_αs/olf_ immunoreactivity in the cytoplasm, dendrites, and apical structures as well as in the axons originating from the *fila olfactoria* in the *lamina propria*. This distribution of the staining is not the same in other fish species that have been examined. For example, in goldfish (*Carassius auratus*), *G*_αs/olf_ was limited to the ciliary surface of the olfactory epithelium, the cell membrane and the axons in the *fila olfactoria* (Hansen et al. 2004). In contrast, in catsharks (*Scyliorhinus canicula)* and sharks in general*,* the *G*_αs/olf_ immunoreactivity was absent due to the lack of ciliated neurons (Ferrando et al. 2009). We could also attribute the different staining patterns in ciliate olfactory neurons of Delta Smelt to the cross reactivity of the antibody with perhaps a different protein, or that we used formalin fixed paraffin embedded sections, which could change antigenic properties of the target protein.

In addition to ciliated neurons, we identified a variety of other cell types indicative of a functional sense of smell. These included microvillous neurons and crypt neurons. Microvillous neurons were similar to those described in other fish species, in that their microvilli are shorter than cilia and lack microtubules (Zippel et al. 1996; Hansen and Zielinski 2005). In goldfish (*Carassius auratus*), it has been suggested that microvillous cells play a role in pheromone (Zippel et al. 1996), and amino acid detection (Speca et al. 1999), while ciliated neurons are involved in the recognition of food odors, amino acids (Zippel et al. 1996) and nucleotides (Hansen et al. 2003). Additionally, in male zebrafish (*Danio rerio*), ciliated OSNs have been shown to detect prostaglandin F_2α_ involved in mating behavior (Yabuki et al. 2016)_._ Whether these properties are similar in Delta Smelt is unknown and warrants further investigation.

Interestingly, we also observed populations of crypt neurons surrounded by sustentacular cells. Crypt neurons have frequently been described in zebrafish (*Danio rerio*) (Hansen and Eckart 1998) and other fish species (Hansen and Finger 2000). Using calcium imaging and confocal microscopy, crypt neurons have been demonstrated to respond to gonadal extracts and hormones in rainbow trout (*Oncorhynchus mykiss*) and to kin odors in zebrafish (*Danio rerio*), suggesting they are involved in mediating reproductive signaling and kin recognition (Hansen and Finger 2000; Biechl et al. 2016; Bazaes and Schmachtenberg 2012; Hamdani el and Doving 2006).

Whereas in zebrafish olfactory epithelium, S-100 immunoreactivity tends to be exclusively restricted to crypt cells (Germanà et al. 2004; Lazzari et al. 2017), in Delta Smelt, we observed staining in all sensory neuronal subpopulations (crypt cells, ciliated OSNs and microvillous neurons). In addition, the calretinin stain identified sensory neurons that ran continuously along the epithelial surface, implying that they were more widespread in Delta Smelt than has previously been reported in zebrafish (*Danio rerio*) (Bettini et al. 2016), and goldfish (*Carassius auratus*) (Hansen et al. 2004). Finally, we observed cells with rod-shaped apical modifications. This cell type was originally described by Bannister (1965) in the minnow (*Phoxinus phoxinus*), and later in the goldfish (*Carassius auratus*) (Ichikawa and Ueda 1977) as a cell type that lacks both cilia and microvilli, but instead has a naked single rod that extends from the surface. On the other hand, Moran et al. (1992) suggested that such rod-like structures were fixation artifacts caused by the fusion of cilia into a single structure. Using fluorescence and electron microscopy, these cells have, however, recently been confirmed to be an actin rich cell type in larval zebrafish (*Danio rerio*) (Cheung et al. 2020). Whether or not this type of cell has a sensory function is unknown.

Other sensory neurons described in the olfactory epithelium of fish are the Kappe neurons (Ahuja et al. 2014) and the pear-shaped neurons (Wakisaka et al. 2017). These cell types have similar morphologies and distributions to crypt cells, but express different markers. The kappe neurons are *G*_O_ immunoreactive and are positioned apically in the olfactory epithelium (Ahuja et al. 2014). On the other hand, pear-shaped neurons have been shown to detect adenosine and ATP and are morphologically similar to crypt neurons. We were not able to identify these neurons in Delta Smelt. Immunostaining for *G*_O_ was inconclusive in formalin fixed paraffin embedded sections. More targeted studies using, frozen sections, immunofluorescence, and in situ hybridization would likely be needed to definitively confirm whether these cell types occur in Delta Smelt.

### Cytology of non-sensory supporting cells

In the Delta Smelt olfactory rosette, non-sensory cells were also similar to those described in other fish species. We identified morphologically distinct populations of both ciliated and secretory sustentacular cells. The nuclei of these cells were mostly located basally within the epithelium. This basal position suggests an inverted morphology, as has been demonstrated in zebrafish (*Danio rerio*) (Demirler et al. 2020). The granules in secretory sustentacular cells were ultra-structurally different from mucin vesicles in goblet cells and were more electron dense. It has been suggested that these secretory granules produce the mucopolysaccharides that form the mucinous layer that coats the olfactory epithelium (Zeiske et al. 1992; Hansen and Zielinski 2005). These cells are thus thought to serve the same lubrication function as the Bowman’s gland found in the olfactory epithelium of other vertebrates (e.g., Chinese softshell turtles, *Pelodiscus sinensis*) (Getchell and Getchell 1992; Nakamuta et al. 2016). In goldfish (*Carassius auratus*) and zebrafish (*Danio rerio*), sustentacular cells have been reported to contain clear electron lucent vesicles in the apical domain (Byrd and Brunjes 1995; Zippel et al. 1996; Hansen and Eckart 1998), whereas the granules in sustentacular cells from Delta Smelt were more electron dense, consistent with secretory granules.

We also observed a filamentous layer of electron dense material on the ciliary surface of the lamellae. This material likely originated from the mucin strands that formed the mucous layer on the epithelial surface when the fish was alive. This mucous layer has been described in different fish species and is thought to play a role in concentrating odorants before they reach the olfactory neurons (Getchell and Getchell 1992). For example, in fresh water, the amino acids glycine and alanine were more readily soluble in the mucus phase (partition coefficient less than 1) than in the aqueous phase of the mucus layer, which could serve to concentrate odorants, prior to detection at the level of the epithelium (Rygg et al. 2013). Interestingly, in the Delta Smelt, we observed that sustentacular cells and olfactory neurons were evenly distributed throughout the lamellar surface. If the layer of mucus is also uniformly distributed over a diffuse population of olfactory neurons, the chance of fish detecting an odorant might be enhanced (Rygg et al. 2013).

We observed a rich network of blood vessels in the olfactory rosette *lamina propria*, which suggests that the olfactory system of Delta Smelt is functional and that an ample blood supply is available to support the increased energetic demands of olfactory neurons and sustentacular cells (Klimenkov et al. 2020). The abundant mitochondria in sustentacular cells and olfactory neurons would require a considerable amount of oxygen to produce energy, and a well-developed capillary network would help in this function. These observations provide further evidence that Delta Smelt have a functional sense of smell rather than a vestigial structure.

### Cytology of immune cells

The capsule in which the rosette sits was lined by an epithelium characteristic of a mucosal surface committed to immune surveillance and secretion. The abundance of goblet cells and secretory granules suggest a secretory function, and an abundant population of immune resident cells is a characteristic of fish mucosal surfaces including the sensory and non-sensory epithelium (Gomez et al. 2013). For example, Tacchi et al. (2014) found that rainbow trout (*Oncorhynchus mykiss*) have a diffuse population of lymphoid cells (predominantly B-lymphocytes) that forms the Nasopharynx-Associated Lymphoid Tissue (NALT), in the sensory and non-sensory epithelium, and in the *lamina propria* of the olfactory rosette. Together, the mucosal immune cells and the sensory neurons have been shown to trigger an effective immune response against pathogens (Sepahi et al. 2019; Das and Salinas 2020). Cells in the capsular epithelium of Delta Smelt resembled lymphocytes in terms of their shape and nuclear morphology. However, specific immunohistochemical studies would be needed to confirm their identity. In other teleosts, rodlet cells have been observed in various tissues and mucosal surfaces (Reite 2005), including the olfactory epithelium (Hansen and Zielinski 2005). The rodlet cells are thought to play a role in parasitic infections, acting mainly by secreting the products in the crystalline core of rods to the extracellular space (Reite and Evensen 2006). These histological features suggest an immunological function of the sensory and non-sensory epithelium of Delta Smelt, leaving this question open for further study.

## Conclusions and future directions

We demonstrated that the morphological features of the Delta Smelt olfactory rosette suggest that these fish have a well-developed sense of smell. The variety and complexity in cell structure and morphology indicates a diverse function and specialization of olfactory neurons to detect complex blends of odorants, suggesting that Delta Smelt rely on their sense of smell and that this feature of their sensory ecology has been overlooked. Our morphologic examination suggests that their olfactory system is robust and should be ecologically important for their survival. We are starting to understand the olfactory biology in this endangered fish. In the Anthropocene, a changing environment has been shown to deleteriously impact fish that rely on olfaction for many aspects of their life history (Tierney et al. 2010; Cattano et al. 2019). Thus, studies on basic morphology will inform and give context to research on the pathological effects of contaminant exposure and broadens our understanding of the olfactory biology of this endangered fish.

## Supplementary Information

Below is the link to the electronic supplementary material.

Supplementary file1 (PDF 2845 kb)

## Data Availability

Supplementary material is provided in the online version of this article. All the data that support the findings are provided within the manuscript.
