# Supplementary material for: Gross morphology, histology, and ultrastructure of the olfactory rosette of a critically endangered indicator species, the Delta Smelt, Hypomesus transpacificus
Source: J Comp Physiol A Neuroethol Sens Neural Behav Physiol. 2021 Jun 22;207(5):597–616. doi: 10.1007/s00359-021-01500-7 (PMC8408092; doi:10.1007/s00359-021-01500-7)
Supplement: Supplementary file 1 — Supplementary file1 (PDF 2845 kb) [file 359_2021_1500_MOESM1_ESM.pdf]

Online Resource

Gross morphology, histology, and ultrastructure of the olfactory rosette of a critically endangered indicator species, the Delta Smelt, *Hypomesus transpacificus*

Triana-Garcia, P, A[1,2]; Nevitt, G, A[3]; Pesavento J, B[4]; Teh, S, J[1].

[1] Integrative Pathobiology Graduate group and Aquatic Health Program, School of Veterinary Medicine, Department of Anatomy, Physiology and Cell Biology, University of California, Davis, California, United States of America.

[2] Grupo de Investigación en Sanidad de Organismos Acuáticos, Instituto de Acuicultura de los Llanos, Universidad de los Llanos, Villavicencio, Meta, Colombia.

[3]Department of Neurobiology, Physiology and Behavior, University of California, Davis, California, United States of America.

[4]California Animal Health & Food Safety Laboratory, School of Veterinary Medicine, University of California, Davis, California, United States of America.

ORCID ID

Triana-Garcia, P, A: https://orcid.org/0000-0002-8079-1097

Nevitt, G, A: https://orcid.org/0000-0002-0346-2126

Pesavento,J,B: https://orcid.org/0000-0002-4642-8593

Teh, S, J: https://orcid.org/0000-0002-0587-3711

Corresponding author

Pedro Alejandro Triana Garcia, Department of Anatomy, Physiology and Cell Biology, School of Veterinary Medicine, University of California, VM3B, 3203, 1089 Veterinary Medicine Dr, Davis, CA 95616

Email: trianagarcia@ucdavis.edu

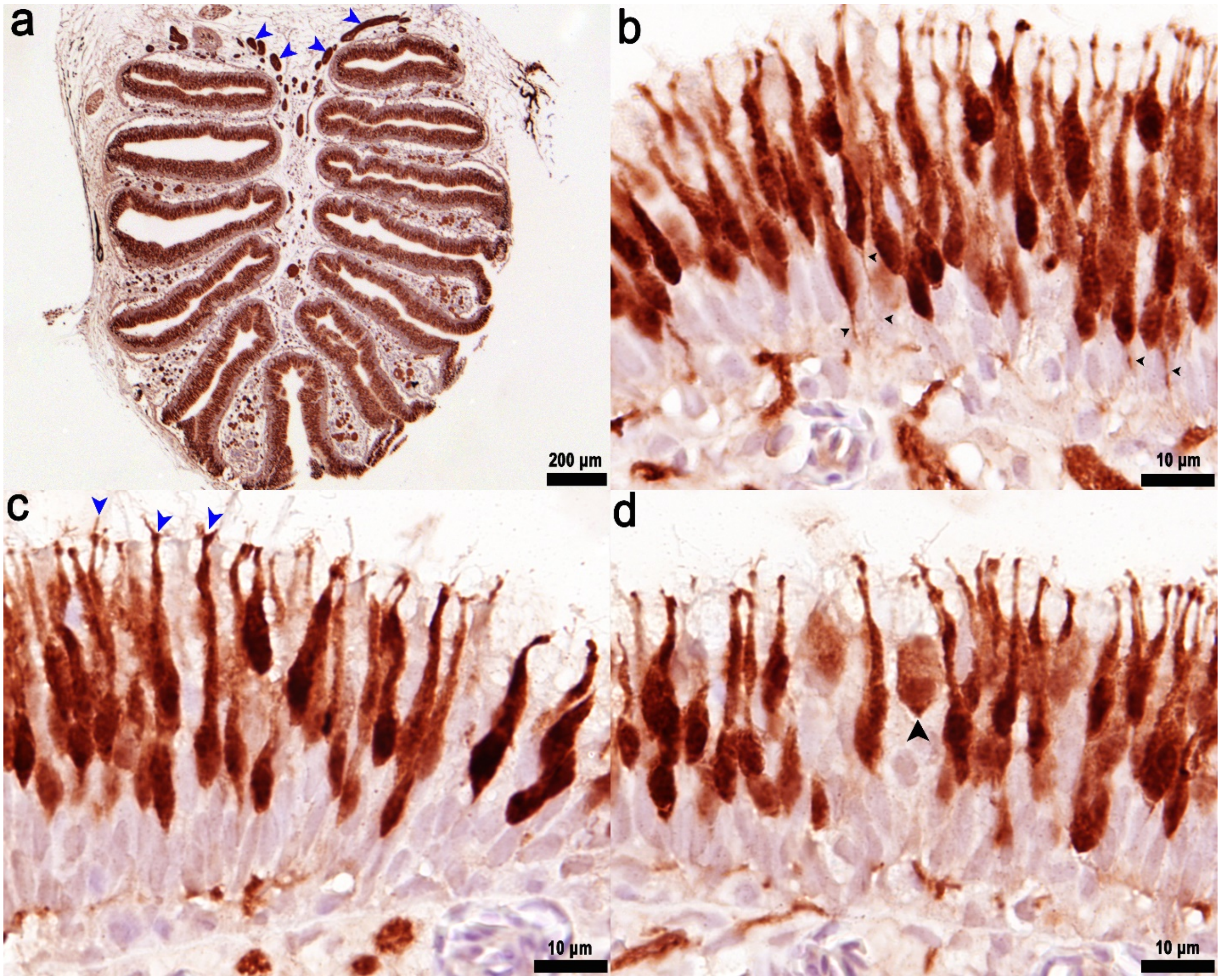

Online resource Fig. 1 S-100 immunolabeling highlighting the distribution of a heterogeneous population of sensory neurons in the olfactory rosette of Delta Smelt. **a** Whole olfactory rosette showing the distribution of sensory neurons. Note the strong immunolabeling of nerve bundles in the lamina propria of the rosette (blue arrowheads); 40X, light microscopy photomicrograph. **b** Higher magnification of sensory neurons. Axons (black arrowheads) can be seen projecting from olfactory neurons towards the lamina propria; 400X, light microscopy photomicrograph. **c and d.** Ciliated sensory neurons (**c,** blue arrowheads) and crypt neurons (**d,** black arrowhead) are similarly immunolabeled; 400X, light microscopy photomicrograph.

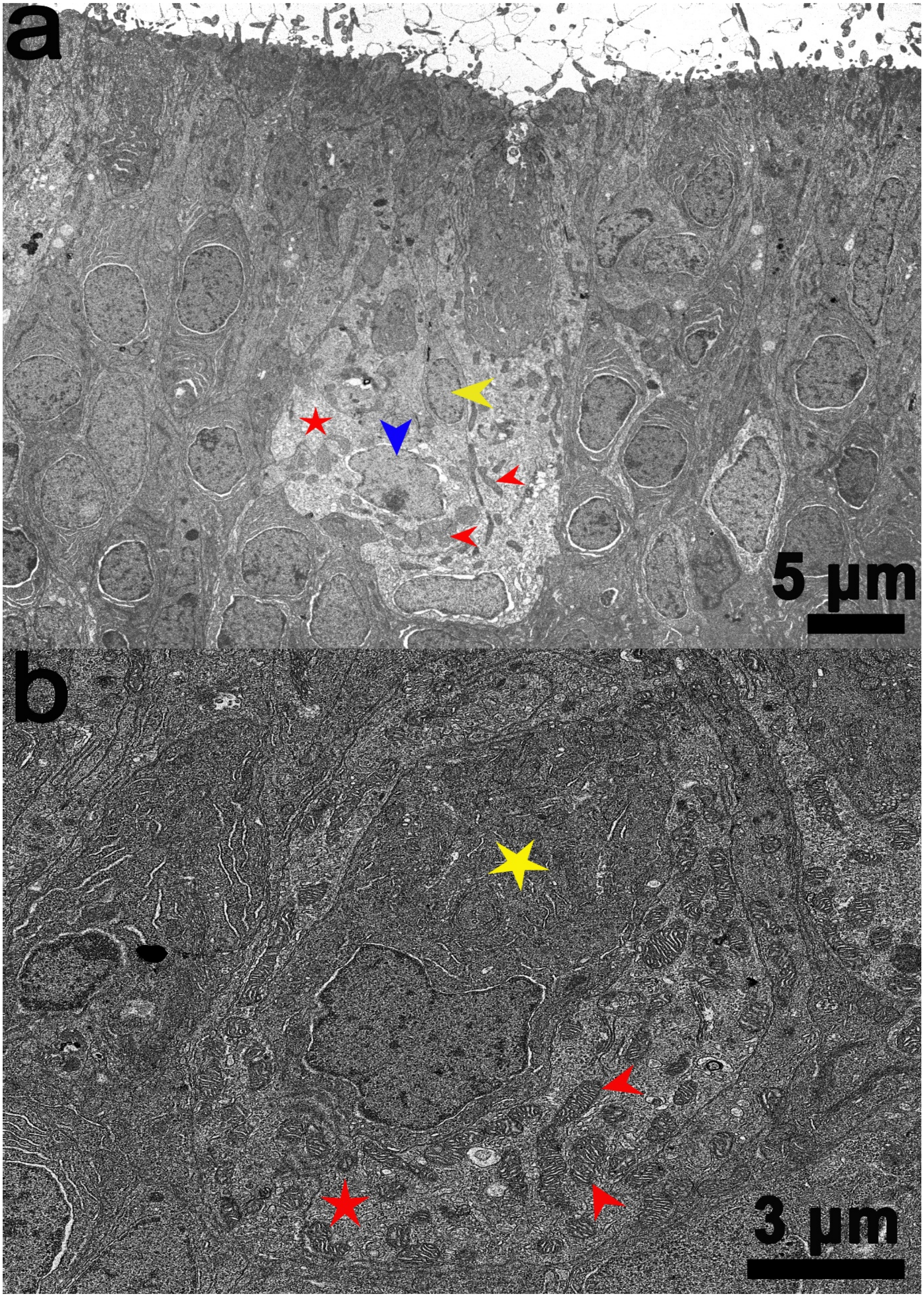
a
5 µm
b
3 µm

Online resource Fig. 2 TEM micrographs of sustentacular cells surrounding a sensory neuron. **a** A sustentacular cell with abundant smooth endoplasmic reticulum (red star) and mitochondria (red arrow heads) is shown. The blue arrowhead points to the nucleus. The yellow arrowhead points to the nucleus of an associated sensory neuron 1250X, TEM. **b** Detail of sustentacular cells (red star) and an associated sensory neuron (yellow star). Note the abundant mitochondria (red arrowheads); 1250X, TEM.
